# Continuous endoglin (CD105) overexpression disrupts angiogenesis and facilitates tumor cell metastasis

**DOI:** 10.1007/s10456-019-09703-y

**Published:** 2020-01-03

**Authors:** Claudia Ollauri-Ibáñez, Elena Núñez-Gómez, Cristina Egido-Turrión, Laura Silva-Sousa, Elena Díaz-Rodríguez, Alicia Rodríguez-Barbero, José M. López-Novoa, Miguel Pericacho

**Affiliations:** 1 Renal and Cardiovascular Research Unit, Department of Physiology and Pharmacology, University of Salamanca, and the Biomedical Research Institute of Salamanca (IBSAL), Edificio Departamental, Campus Miguel de Unamuno, 37007 Salamanca, Spain; 2 Instituto de Biología Molecular Y Celular del Cáncer. CSIC, IBSAL and CIBERONC, Salamanca, Spain

**Keywords:** Endoglin, CD105, Angiogenesis, Cancer, Metastasis

## Abstract

Endoglin (CD105) is an auxiliary receptor for members of the TFG-β superfamily. Whereas it has been demonstrated that the deficiency of endoglin leads to minor and defective angiogenesis, little is known about the effect of its increased expression, characteristic of several types of cancer. Angiogenesis is essential for tumor growth, so high levels of proangiogenic molecules, such as endoglin, are supposed to be related to greater tumor growth leading to a poor cancer prognosis. However, we demonstrate here that endoglin overexpression do not stimulate sprouting or vascularization in several in vitro and in vivo models. Instead, steady endoglin overexpression keep endothelial cells in an active phenotype that results in an impairment of the correct stabilization of the endothelium and the recruitment of mural cells. In a context of continuous enhanced angiogenesis, such as in tumors, endoglin overexpression gives rise to altered vessels with an incomplete mural coverage that permit the extravasation of blood. Moreover, these alterations allow the intravasation of tumor cells, the subsequent development of metastases and, thus, a worse cancer prognosis.

**Electronic supplementary material:**

The online version of this article (10.1007/s10456-019-09703-y) contains supplementary material, which is available to authorized users.

## Introduction

Angiogenesis is the formation de novo of blood vessels from preexisting ones in response to several stimuli, mainly hypoxia. Thus, angiogenesis responds to tissue requirements of blood supply, being therefore a key process in several pathologies such as tumor growth [1, 2]. During angiogenesis, preexisting endothelial cells (ECs) perform highly coordinated morphogenic events that include basement membrane degradation, EC sprouting and branching, vessel lumen formation, vessels anastomosis and mural cell recruitment, resulting in a new vascular network that provides blood to the hypoxic tissue [3, 4].

Endoglin (CD105) is an auxiliary receptor for several members of the TFG-β superfamily of cytokines. There is a substantial amount of evidence that relates endoglin to angiogenesis. On the one hand, endoglin expression is increased in the endothelium during active angiogenesis [5], specifically at the angiogenic edge, where sprouting takes place [6–8]. On the other hand, deficiency in endoglin expression is responsible for hereditary hemorrhagic telangiectasia type-1 (HHT-1), a disease characterized by vascular malformations [9]. In experimental models, the lack of or deficiency in endoglin produces minor and defective angiogenesis due to alterations of ECs physiology [7, 10–12]. However, the effect of increased endoglin expression, described in pathologies such as cancer, on the function of ECs and angiogenesis has not been deeply studied.

Cancer is a generic term for a large group of diseases characterized by the growth of abnormal cells (tumor cells) beyond their usual boundaries, invading adjoining parts of the body and even spreading to other organs. Tumoral cells are highly proliferative, expansive and have high-metabolic requirements; thus, the oxygen demand soon exceeds the input [13]. For this reason, as described in the 1970s, solid tumors cannot grow more than a few millimeters in the absence of the development of new blood vessels [14]. In tumors, many of the processes that take place during angiogenesis are dysregulated, leading to irregular and chaotic tumor neovessel formation and thus to dysfunctional and more permeable vessels that facilitate the intravasation and homing of tumor cells in other tissues [13, 15].

Because angiogenesis is essential for the growth of tumors, the quantification of microvessel density is a frequent clinical practice to assess tumor prognosis [16–18]. More specifically, a high number of endoglin-positive microvessels detected by immunohistochemistry has been associated with poor prognosis in some solid tumors [19, 20]. Moreover, enhanced endoglin expression in tumor microvessels is a better outcome predictor for some cancer types than the levels of other angiogenesis-related molecules [21, 22]. In addition, treatment with the anti-endoglin monoclonal antibody TRC105 or with different anti-endoglin shRNAs, siRNAs or miRNAs reduces tumor growth through the reduction of blood vessels inside the tumor [23]. All these observations have led to the assumption that the worse prognosis of tumors with high levels of endoglin is due to greater angiogenesis and, therefore, greater tumor growth, although this hypothesis has not been experimentally demonstrated. Recently, it has been shown that anti-endoglin therapy reduces the generation of metastases [24, 25]; however, the exact mechanisms by which endoglin could contribute to the generation of metastases have not been reported.

Here, we demonstrate that continuous endoglin overexpression does not enhance angiogenesis rate. However, it produces alterations throughout the course of angiogenesis process that prevent blood vessels from maturing and stabilizing. Thus, contrary to what had been hypothesized, endoglin overexpression does not result in an increase in tumor growth or tumor vascularization but results in even poorer quality tumor vessels, facilitating the intravasation and metastasis of tumor cells. For this purpose, we used transgenic mice-overexpressing endoglin (*ENG*^+^) in a C57BL/6J background, with C57BL/6J mice as controls (WT) [26], and several in vitro approaches to study angiogenesis and the specific events that take place during blood vessel formation.

## Materials and methods

The methodological procedures are detailed in the “Supplementary methods” section.

### Mice

All animal procedures were conducted in strict compliance with the European Community Council Directive (2010/63/EU) and Spanish legislation (RD1201/2005 and RD53/2013). The protocols were approved by the University of Salamanca Ethical Committee. Animal selection was genotype based and no randomization or blinding was performed. Animals were housed under specific pathogen-free conditions in University of Salamanca facilities (ES-119-002001 SEARMG), in a temperature-controlled room with 12-h light/dark cycle and reared on standard chow and water provided ad libitum. The generation and characteristics of the transgenic mice ubiquitously overexpressing endoglin (*ENG*^+^) was performed by microinjection of a pCAGGS vector containing the complementary DNA sequence (cDNA) of endoglin (Supplementary Fig. 1A) in the fertilized egg of CBAxC57BL/6J mice [26]. Wild-type C57BL/6J mice (WT) were used as controls. No differences were found in the litter size, in the weight of the pups or in the weight between 1 and 6 months (Supplementary Fig. 1B–D). Human endoglin overexpression was demonstrated in several tissues by qPCR (Supplementary Fig. 1E). For animal anesthesia, 2% isoflurane in oxygen was used. During recovery from the anesthesia, heat was provided and, when necessary, a dose of buprenorphine (0.05 mg/kg) was subcutaneously administered. Animals were sacrificed by CO_2_ inhalation or cervical dislocation, depending on the age and the experiment requirements.

### Cells and cell cultures

MLECs were isolated and purified following “Mouse Lung Endothelial Cell (MLEC) culture”. Then, they were cultured in Petri dishes coated with 0.1% gelatin (Sigma-Aldrich) supplemented with 0.01% collagen (Corning) and 0.01% fibronectin (Sigma-Aldrich) with ‘MLEC culture medium’, i.e., Advanced-DMEM F-12 (Thermo Fisher Scientific) supplemented with 20% FBS, 50 U/mL penicillin–streptomycin, 2 mM glutamine (Sigma-Aldrich), 30 μg/mL endothelial cell growth supplement (ECGS) (Generon) and 100 μg/mL bovine heparin (Sigma-Aldrich). MLECs between passages 3 and 6 were used for the experiments. The human EC line EA.hy926 was supplied by the ATCC and cultured in DMEM, 10% FBS and 50 U/mL penicillin–streptomycin. EA.hy926 cells were stably infected (“Cellular infection”) with a vector containing the sequence encoding human endoglin (*ENG*^+^) (Supplementary Fig. 1A) or an empty vector (*Mock*). HBVPs were supplied by ScienCell™ and cultured in pericyte medium (PM), 2% FBS, 1% pericyte growth supplement and 1% penicillin/streptomycin (ScienCell™). HBVPs were cultured in Petri dishes coated with 2 μg/cm^2^ of poly-l-lysine (Sigma-Aldrich). LLC cells, provided by Dr. Aliño (Department of Pharmacology, University of Valencia, Spain), were cultured in Dulbecco’s modified Eagle’s medium (DMEM) (Thermo Fisher Scientific) supplemented with 10% fetal bovine serum (FBS) (Thermo Fisher Scientific) and 50 U/mL penicillin–streptomycin (Thermo Fisher Scientific). GFP-LLC cells were generated according to the above-mentioned cell infection protocol. All cell lines were maintained in 90% RH, 5% CO_2_ atmosphere at 37 °C.

### Endothelial cell proliferation and migration

Endothelial cell proliferation was evaluated by “Direct cell proliferation”. Moreover, the bromodeoxyuridine (BrdU) incorporation ELISA kit (Ref. 11647229; Roche) was used following manufacturer’s instructions. 10^3^ MLEC or EA.hy926 ECs per well were plated in 96-well plates. After 24 h, cells were incubated with BrdU (4 h for EA.hy926 ECs and 24 h for MLECs). 100 μL of substrate solution was added and, after 5 to 30 min, fluorescence at 370 nm was read. Cell motility was assessed following “Wound healing migration assay (Scratch assay)”, while migration and ECM invasion were assessed following “Transwell assay”.

### In vitro angiogenesis assays

Endothelial cell organization was evaluated by “Capillary-like structures in Matrigel^®^”. Moreover, pericyte recruitment by “Co-culture of ECs and pericytes in Matrigel^®^” and “Pericyte adhesion” to an endothelial monolayer were assessed. Cell stability was also evaluated in vitro*.* For this aim, two 35-mm culture dishes were seeded with 3 × 10^4^*ENG*^+^ or WT MLECs. Cells from one dish were collected for RNA isolation after 8 h; cells from the other dish were cultured up to 100% confluence prior to RNA isolation.

### Ex vivo and in vivo angiogenesis assays

Sprouting was also evaluated by “Aortic ring assay”. “Mouse hindlimb ischemia” was performed and perfusion was evaluated in the ischemic and non-ischemic limbs using Doppler Laser Moor LDLS (Moor Instruments) on days 1, 3, 5, 7, 14, 21 and 28 after ischemia. For histological analysis, the soleus muscles were collected 14 days after surgery. “Direct In Vivo Angiogenesis Assay (DIVAA™)” was used for quantification of the blood-invaded distance and the content in endothelial cells. The low number of cells inside the tubes made RNA extraction and histological studies impossible. Therefore, an analog to DIVAA, namely “Plugs of Matrigel^®^”, was used. Retinas from P6 and P17 pups were dissected and vascularization was evaluated by immunofluorescence and by mural and endothelial marker expression following “Retina dissection and immunostaining” protocol. Immunofluorescence antibodies: FITC-lectin B4 (Ref. 3450-048-FL; Trevigen), anti-VE-cadherin (Ref. 555289; BD Pharmingen), anti-Ki67 (Ref. Ab15580; Abcam), anti-NG2 (Ref. AB5320; Merck) and anti-human endoglin (Ref. ab114052; Abcam). Three-dimensional rendering of confocal Z-stacks was performed with Fiji software. The areas occupied by the pericytes were quantified using Adobe Photoshop CS6. The “patching algorithm” for MATLAB, provided by Dr. Bentley (Department of Immunology, Genetics and Pathology, Uppsala University, Sweden), was used for VE-cadherin patterning quantification.

### Tumor angiogenesis assays

Tumors were generated following a “LLC cell tumor xenograft model”. 10 days after the injection “tumor hemoglobin and DNA measurement” was realized. “Lung metastases” and “circulating GFP-LLC cells” were quantified in mice injected with GFP-LLCs.

### Histological tissue analysis

Tumors and limbs (soleus muscle) intended for histological analysis were fixed in 4% PFA for 24 h, dissected and embedded in paraffin. 3-μm sections were stained by hematoxylin–eosin (standard protocol) or processed for immunohistochemistry. Immunohistochemistry was carried out using a DISCOVERY ULTRA system (Roche) and the DISCOVERY ChromoMab DAB kit (RUO) (Ref. 760-159; Roche). Sections were incubated with the primary antibody anti-human endoglin (Ref. ab219362; Abcam), anti-Pecam1 (Ref. ab28364, Abcam) or anti-αSMA (Ref. ab5694; Abcam) for 1 h and with OmniMap™ Anti-Rb HRP (Ref. 760-4311; Roche) for 12 min or 1 h with anti-rabbit Ig secondary antibody (Ref. 170-6515; Bio-Rad). For tumors, 20 images per slide were analyzed for the area occupied by erythrocytes and the number of vessels using Fiji software. For muscles, 25 to 30 images per slide were analyzed for the number of vessels and vessel diameter with Fiji. Partial or total coverage of vessels with α-SMA in tumors and muscles was manually determined in each vessel by a researcher, without knowing if the tissue was from a WT or *ENG*^+^ mouse. Plugs of Matrigel^®^ were fixed in Somogyi solution at 4 °C for 24 h and then transferred to 30% sucrose and 0.05% sodium azide in PBS 1X for 24 h and subsequently embedded in OCT. Sections (15-μm thick) were fixed in 4% PFA for 5 min; blocked in 5% donkey or goat serum, 1% BSA, 0.5% Tween^®^ 20 in PBS for 2 h; and incubated with anti-Pecam1 (Ref. 550274; BD Pharmingen) and anti-NG2 (Ref. AB5320; Merck) overnight. The sections were incubated with secondary antibodies (Thermo Fisher Scientific) for 4 h. The slides were mounted with Prolong^®^ Gold Antifade (Thermo Fisher Scientific) and observed using an Axiovert 200 M fluorescence microscope (Nikon). The pericyte coverage of plug vessels was evaluated by assigning a score (1: low coverage–3: high coverage) to each vessel in the largest possible number of photos for each line.

### Protein expression

Protein expression was evaluated in EA.hy926 cells and MLECs by “Western blot”, “Flow cytometry” and “Cellular immunofluorescence”. Antibodies for western blot: anti-human endoglin (Ref. H300; Thermo Fisher Scientific), anti-mouse β-actin (Ref. A5441; Sigma-Aldrich). Antibodies for flow cytometry: PE mouse anti-human CD105 (Ref. 560839; BD Biosciences). Antibodies for immunofluorescence: anti-human endoglin hybridoma TEA1/58.1 (provided by Dr. Sánchez-Madrid, CNIC, Spain), anti-mouse VE-cadherin (Ref. ab33168; Abcam) and secondary antibody anti-mouse IgG AlexaFluor568^®^ (Ref. A10037; Thermo Fisher Scientific) were used. The “patching algorithm” for MATLAB was also used for VE-cadherin patterning quantification in EA.hy926 ECs.

### Gene expression

RNA from tumors, cultured cells, plugs of Matrigel^®^ and retinas were isolated using NucleoSpin^®^ RNA (Ref. 740955; Macherey-Nagel) according to manufacturer’s instructions. RNA from sprouts of aortic rings was extracted using NucleoSpin^®^ RNA XS (Ref. 740.902; Macherey–Nagel). In samples with high-Matrigel^®^ content (plugs and sprouts) manufacturer’s instructions for fibrous tissues were followed. cDNA synthesis was performed with iScript RT Supermix (Ref. 1708841; Bio-Rad). cDNA from sprouts and plugs was preamplified with ssoAdvanced PreAmp Supermix (Ref. 1s725160; Bio-Rad) and Prime PCR Preamp Assays 2× (Bio-Rad), listed in Supplementary Table 1. For qPCR, Supermix iQTM SYBR^®^ Green (Ref. 170–8882; Bio-Rad) and an iQTM 5 System (Bio-Rad) were used. Gene expression results were normalized to ribosomal protein S13 (Rps13) and β-actin (Actb) for mouse genes, to glycealdehyde-3-phosphate dehydrogenase (Gapdh) for human genes and to EC markers when we approximated the gene expression to EC content within the tissue. The primers used are listed in Supplementary Tables 1 and 2.

### Data presentation and statistical analysis

All images shown are representative and all data, except those obtained by qPCR or the scores of VE-cadherin junctions and mural cell coverage, are expressed as the mean ± SEM. qPCR results are represented in box plots that show the median and the 25–75th percentiles, with whiskers showing the 10–90th percentiles. Scores of VE-cadherin junctions and mural cell coverage are represented as a frequency distribution and a chi-squared test was applied for statistical analysis. For the in vivo experiments, at least eight animals were used per group, when possible. In vitro experiments were repeated at least three times. The D’Agostino–Pearson normality test was applied to the datasets prior to statistical comparations. The Kolmogorov–Smirnov test was used for small datasets. For normally distributed datasets, Student’s *t* test was used. The Mann–Whitney *U* test was used as the nonparametric test. Two-way repeated measures ANOVA was used to assess the differences between groups in the time-course experiments. Sidak’s post hoc test was used after ANOVA. All analyses were performed with Graph Pad 6 software.

## Results

### An excess of endoglin in ECs reduces proliferation while promoting migration and extracellular matrix invasion

EC proliferation and migration towards angiogenic stimuli are key highly regulated events during sprouting angiogenesis [27]. Endoglin deficiency results in decreased EC migration and proliferation in vitro and an impairment of in vivo angiogenesis [10]. The effects of endoglin overexpression on these cellular events were assessed in vitro using EA.hy926 ECs (*ENG*^+^ and Mock) and MLECs isolated from *ENG*^+^ and WT mice (Supplementary Fig. 2A–E).

**Fig. 1:**
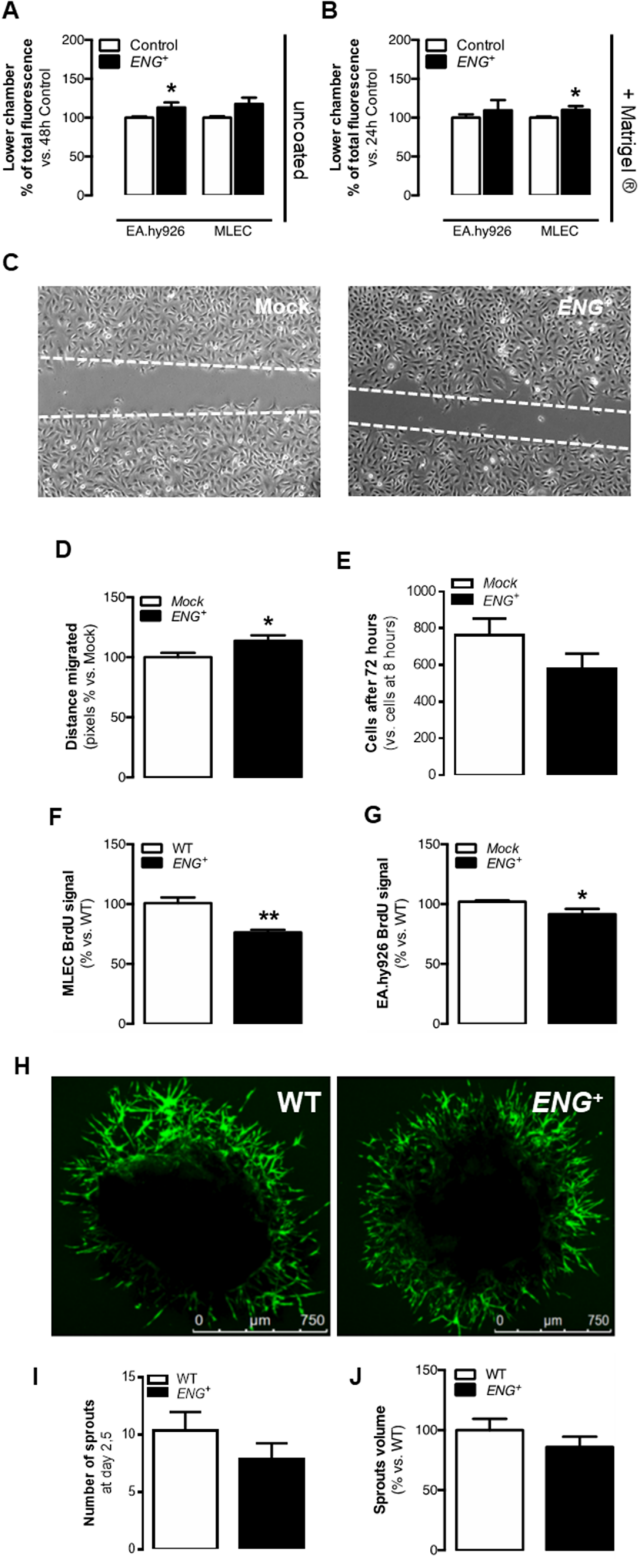
Permanent endoglin overexpression modifies EC physiology in vitro but does not alter sprouting. **a** Quantification of EC migration through the uncoated transwell with a VEGF gradient after 48 h for EA.hy926 and MLEC cells [*n*(Mock) = 3, *n*(*ENG*^+^) = 3; *n*(WT) = 3, *n*(*ENG*^+^) = 3; *p* (EA.hy926) = 0.0297, *p* (MLEC) = 0.0642]. **b** Quantification of EC migration through the Matrigel^®^-coated transwell with a VEGF gradient after 24 h for EA.hy926 and MLEC cells [*n*(Mock) = 3, *n*(*ENG*^+^) = 3; *n*(WT) = 3, *n*(*ENG*^+^) = 3; *p* (EA.hy926) = 0.4453, *p* (MLEC) = 0.0344]. **c** EA.hy926 scratch closure after 14.5 h in culture. **d** Quantification of the distance migrated by EA.hy926 cells though the scratch after 14.5 h [*n*(Mock) = 3, *n*(*ENG*^+^) = 3; *p* = 0.0250]. **e** Ratio of EA.hy926 cell counts after 72 h vs. after 8 h in culture [*n*(Mock) = 3, *n*(*ENG*^+^) = 3; *p* = 0.2030]. **f** BrdU incorporation after 24 h in MLECs [*n*(WT) = 3, *n*(*ENG*^+^) = 3; *p* = 0.0277]. **g** BrdU incorporation after 4 h in EA.hy926 cells [*n*(Mock) = 3, *n*(*ENG*^+^) = 3; *p* = 0.0272]. **h** FITC-lectin-labeled sprout growth from aortic rings isolated from WT and *ENG*^+^ mice. **i** Number of sprouts grown from aortic rings 2.5 days after the seedtime [*n*(WT) = 42, *n*(*ENG*^+^) = 42;*p* = 0.2920]. **j** Quantification of the volume occupied by sprouts from aortic rings [*n*(WT) = 25, *n*(*ENG*^+^) = 20; *p* = 0.2875]

The ability of EA.hy926 EC and MLEC to migrate towards the angiogenic stimulus VEGF was analyzed using uncoated transwells and EC invasiveness through the extracellular matrix (ECM) using Matrigel^®^-coated transwells. Enhanced endoglin expression is associated with an increase in cell migration towards VEGF in both EA.hy926 ECs and MLECs (Fig. 1a). Similarly, endoglin overexpression increases ECM invasion in ECs (Fig. 1b). Moreover, cell motility was analyzed by the scratch assay in EA.hy926 EC monolayers in culture. The results showed that the overexpression of endoglin in human ECs increased their motility in culture (Fig. 1c, d).

A tendency to reduce proliferation in *ENG*^+^ EA.hy926 ECs compared to Mock cells was also observed by direct cell counts (Fig. 1e). For a more precise analysis of cell proliferation, BrdU incorporation in MLECs and EA.hy926 ECs was studied. The level of BrdU incorporation was significantly lower in *ENG*^+^ MLECs and *ENG*^+^ EA.hy926 ECs than in WT and Mock cells, respectively (Fig. 1f, g).

The effect of endoglin overexpression was also evaluated using the in vitro angiogenesis assay. The EA.hy926 *ENG*^+^ or Mock were used for this assay, in which the cells spontaneously organize to form capillary-like structures in Matrigel^®^. We found no differences in the number of branches or in the average branch length in the structures created by Mock and *ENG*^+^ cells (Supplementary Fig. 2F–H).

**Fig. 2:**
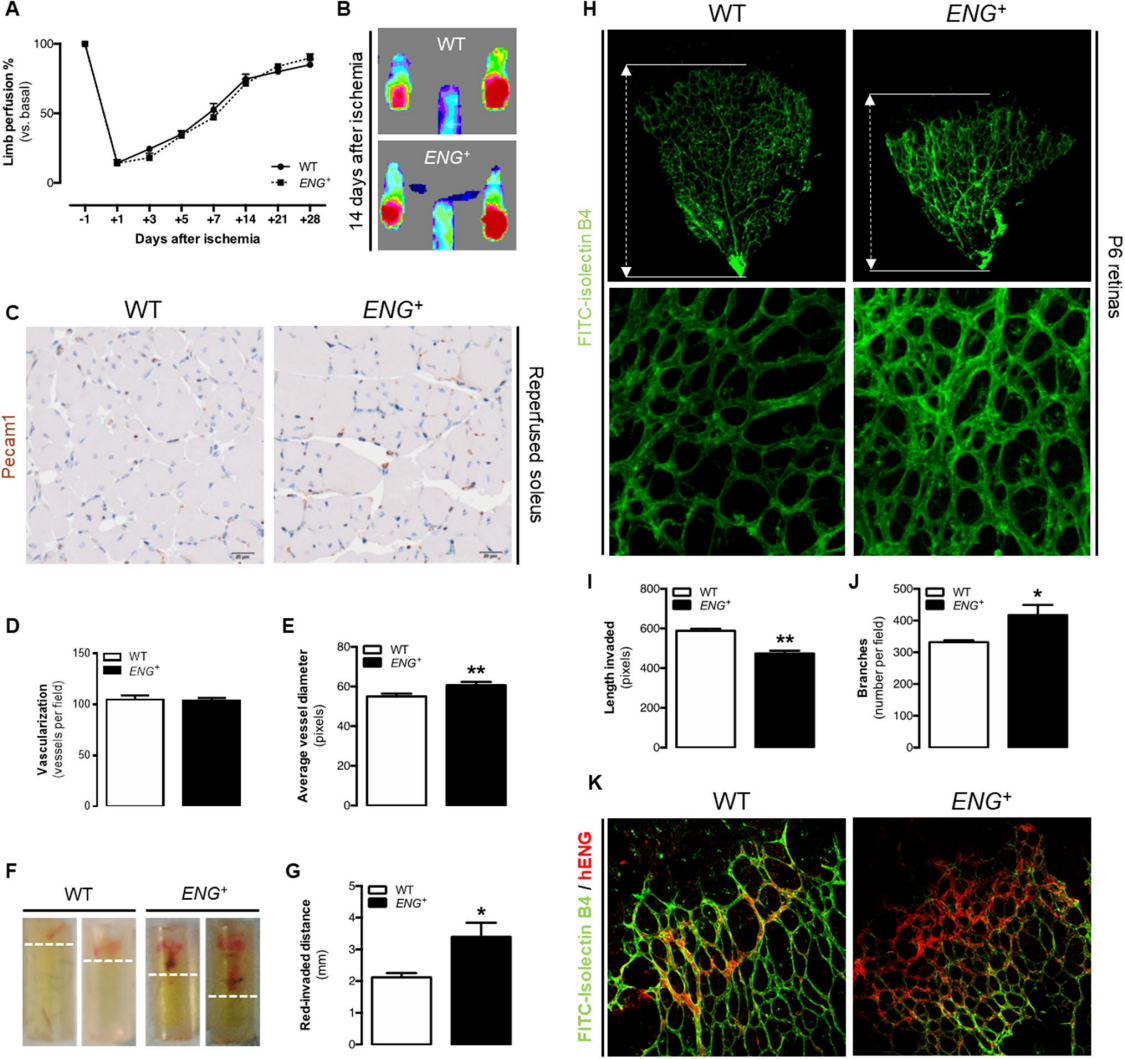
Continuous endoglin overexpression impairs in vivo angiogenesis. **a** Ratio of ischemic to non-ischemic limb perfusion following femoral artery ligation as measured by laser Doppler flow analysis in mice, represented as the percentage of the basal value (before artery ligation) at 1, 3, 5, 7, 14, 21 and 28 days post-ischemia [*n*(WT) = 9, *n*(*ENG*^+^) = 9; *p* = 0.4989]. **b** Laser Doppler images showing mice hindlimb perfusion 14 days after ischemia. **c** Pecam1 immunostaining in the ischemic soleus muscle 14 days post-ischemia. **d** Quantification of the number of Pecam1-positive vessels in the ischemic soleus muscle [*n*(WT) = 4, *n*(*ENG*^+^) = 6; *p* = 0.8204]. **e** Average Pecam1-positive vessel diameter in the ischemic soleus muscle [*n*(WT) = 3, *n*(*ENG*^+^) = 3; *p* = 0.0098]. **f** DIVAA 9 days after implantation in mice, showing blood invasion. **g** Quantification of DIVAA red-invaded distance from the tube end 9 days after implantation [*n*(WT) = 16, *n*(*ENG*^+^) = 28; *p* = 0.0383]. **h** Upper panel: FITC-lectin staining of ECs in the retinal vasculature of p6 pups and representation of the plexus progression. Lower panel: Structure of the vessel plexus of the retina in P6 pups. **i** Quantification of plexus progression in the retinas of P6 pups [*n*(WT) = 7, *n*(*ENG*^+^) = 3; *p* < 0.0001]. **j** Quantification of the ramification of the retinas of P6 pups [*n*(WT) = 7, *n*(*ENG*^+^) = 3; *p* = 0.0227]. **k** Human endoglin (red) and FITC-lectin (green) staining in the angiogenic front of the retinal vasculature of P6 pups. The angiogenic front is oriented towards the upper left of the image in both cases

### Alterations in proliferation and migration caused by endoglin overexpression does not increase sprouting

During sprouting, tip cells are motile and invasive whereas stalk cells are characterized by increased proliferation to support sprout elongation [27]. The results showed above in which *ENG*^+^ ECs are less proliferative, migrate more and have a greater capacity to invade ECM could suggest that endoglin overexpression increases the number or functionality of tip cells. To study the first steps of sprouting, an ex vivo aortic ring angiogenesis assay with samples from *ENG*^+^ and WT mice was performed. In this assay, sprouting from the ring endothelium is induced when it is cultured under proangiogenic conditions. No differences were found neither in the number of tip cells on day 2,5 nor in the volume occupied by the sprouts around *ENG*^+^ and WT rings on day 5 of the assay (Fig. 1h–j). Consistently, qPCR analysis of the expression levels of the specific EC marker *Pecam1* in sprouts showed no significant differences between *ENG*^+^ and WT mice (Supplementary Fig. 3A).

**Fig. 3:**
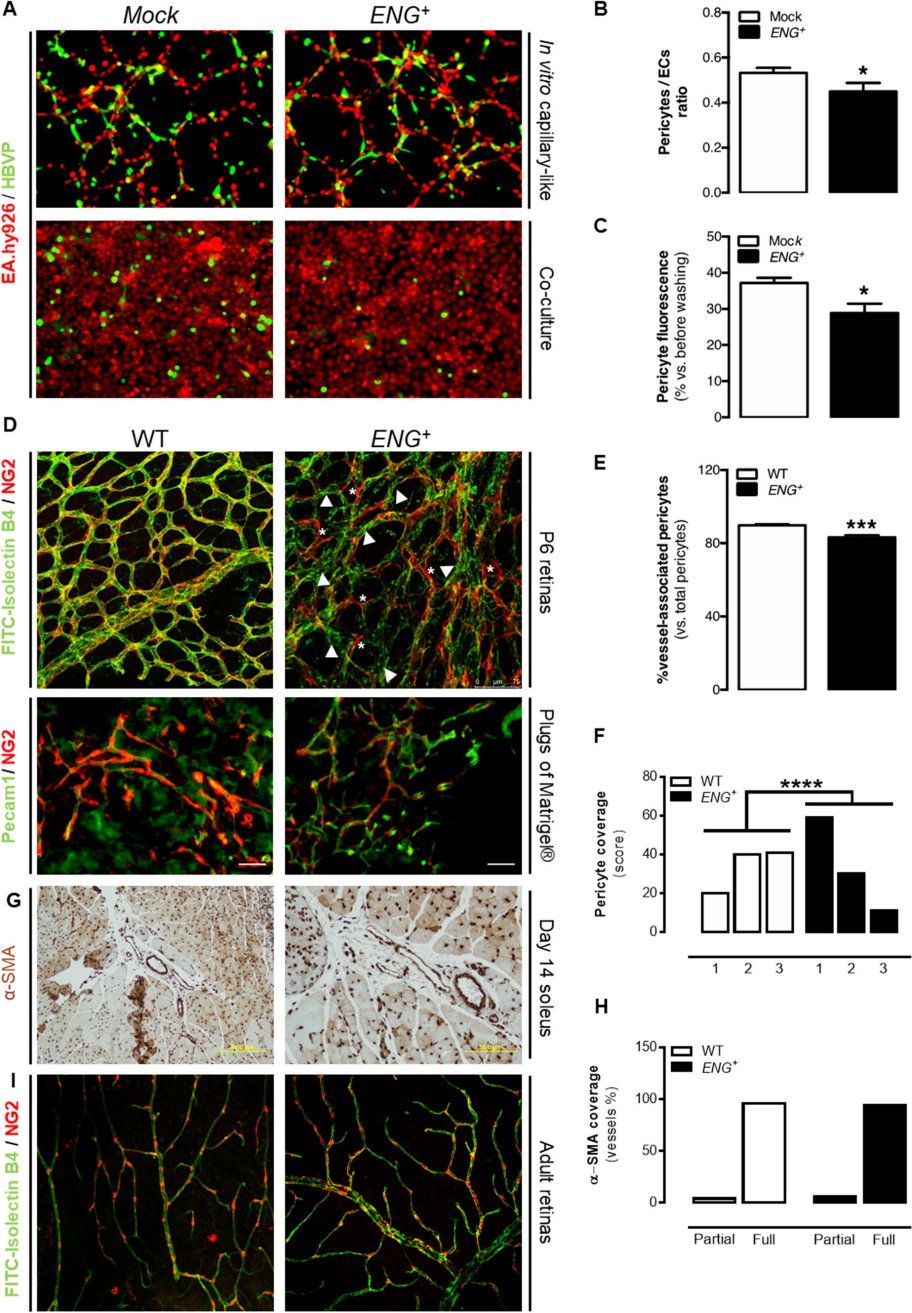
Continuous endoglin overexpression impairs pericyte recruitment in vitro and delays vessel maturation in vivo. **a** Upper panel: Pseudocapillary-like structures formed by EA.hy926 cells cocultured with HBVPs in Matrigel^®^. Lower panel: HBVP attachment to EA.hy926 monolayers in culture. **b** Quantification of the ratio between HBVPs and ECs in pseudocapillary-like structures [*n*(Mock) = 3, *n*(*ENG*^+^) = 3; *p* = 0.0433]. **c** Quantification of the HBVP fluorescent signal over EA.hy926 monolayers [*n*(Mock) = 3, *n*(*ENG*^+^) = 3; *p* = 0.0128]. **d** Upper panel: NG2 (red) and FITC-lectin (green) staining in the retinal vasculature of P6 pups, showing merged signals (yellow) in WT retinas and noncovered endothelium (arrowheads) and mural cells not bound to vessels (asterisk) in *ENG*^+^ retinas. Lower panel: NG2 (red) and CD31 (green) staining in plugs of Matrigel^®^. **e** Quantification of the ratio of pericytes that are bound to the endothelium with respect to the total number of pericytes in the retinal vasculature of P6 pups [*n*(WT) = 4, *n*(*ENG*^+^) = 6; *p* < 0.0001]. **f** Quantification of pericyte recovery in plugs of Matrigel^®^ vessel, where 1 represents low coverage and 3 represents high coverage [*n*(WT) = 4, *n*(*ENG*^+^) = 3; *p* < 0.0001]. **g** α-SMA immunostaining in the ischemic soleus muscle 14 days post-ischemia. **h** Quantification of the ratio of vessels in ischemic soleus muscle that are partially or fully covered by α-SMA immunostaining [*n*(WT) = 3, *n*(*ENG*^+^) = 3; *p* = 1]. **i** NG2 (red) and FITC-lectin (green) staining in the retinal vasculature of adult mice

These results suggest that overexpression of endoglin in ECs may potentiate a tip-like phenotype in vitro, however, this does not result in an increased number of ECs differentiating to tip cells or an enhanced sprouting.

### Sustained endoglin overexpression does not improve post-ischemic reperfusion

To verify whether endoglin overexpression affects the angiogenesis in in vivo models which includes all angiogenic phases, its effect on post-ischemic reperfusion was analyzed. Blood flow in the lower limbs of mice after femoral arterial ligation was measured periodically for 28 days. No significant differences in the reperfusion rates were detected between *ENG*^+^ and WT mice throughout this period (Fig. 2a, b). Furthermore, the structure of the blood vessels was assessed in the soleus muscle after 14 days of ischemia by immunohistochemistry of Pecam1. No differences in vessel number were detected between *ENG*^+^ and WT mice, but dilated vessels observed in *ENG*^+^ mice suggest the presence of alterations in the vessel structure in the reperfused muscle tissue (Fig. 2c–e).

### Constitutive overexpression of endoglin leads to impaired physiological angiogenesis and an altered structure of the new blood vessels

The direct in vivo angiogenesis assay (DIVAA™) consists of the subcutaneous implantation of silicon tubes filled with Matrigel^®^ plus proangiogenic factors into mice. These tubes are invaded by vessels from the mice due to an angiogenic response. The measured distance reached by the red front in each tube was higher in the tubes implanted in *ENG*^+^ mice than in those implanted in WT mice (Fig. 2f, g), which may indicate increased angiogenesis. However, constitutive endoglin overexpression led to a reduced EC content within the tubes 9 days after implantation (Supplementary Fig. 3B). qPCR analysis of the expression levels of the specific EC marker *Pecam1* in the plugs of Matrigel^®^ (analog to DIVAA™) showed no significant differences between *ENG*^+^ and WT mice (Supplementary Fig. 3C). The fact that in *ENG*^+^ mice, the vessels reach a longer distance with no increase in the number of ECs suggests that the red front observed in *ENG*^+^ tubes was not the length invaded by blood-filled vessels but rather extravasated erythrocytes from the defective vessels that moved into the Matrigel^®^.

Another frequently used method to study physiological angiogenesis is the analysis of the development of the retinal vasculature, which in mice takes place between postnatal day 0 (P0) and P8 [28]. Retinas were isolated from P6 *ENG*^+^ and WT pups, and the vessels were identified with FITC-isolectin B4. Structural analysis revealed that compared with WT retinas, *ENG*^+^ retinas showed a lower degree of vascular plexus progression (Fig. 2h, i) and a higher vessel density due to increased ramification (Fig. 2h, j). No differences in the expression levels of the EC marker *Pecam1* were found between retinas from *ENG*^+^ and WT mice (Supplementary Fig. 3D), suggesting that the lower degree of plexus progression is not due to a reduced number of ECs but rather to alterations in angiogenesis-driven vascularization.

Several articles have described a pattern of endoglin expression during retinal angiogenesis. Thus, Barnett et al. has described that endoglin expression is increased in the angiogenic front [6]. Specifically, Jin et al. demonstrated that endoglin expression increased in stalk cells of the retina of WT pups, while in retinas of a mosaic mice cells that overexpressed endoglin were mainly in a tip position [8]. To analyze if our model is consistent with this pattern, human endoglin expression was analyzed by immunofluorescence in pup retinas. In WT retinas, the antibody partially detects murine endoglin, but the staining is limited to that zones where endogenous endoglin expression is increased. As described previously, this increase was located on the angiogenic front, and specifically on stalk cells. In *ENG*^+^ retinas, human endoglin expression is detected in all the retinal vessels, but the staining is stronger just at the edge of the vascular front (Fig. 2k).

### Continuous overexpression of endoglin delays pericyte recruitment to the newly formed capillaries

Endoglin has been reported to play a major role in vessel maturation [29, 30]. Additionally, it has been shown that the RGD sequence of the extracellular domain of endoglin mediates pericyte binding to the ECs [31]. Thus, in vitro mural cell recruitment in a coculture of EA.hy926 ECs and human brain vascular pericytes (HBVPs) in Matrigel^®^ was studied. As discussed above, ECs create capillary-like structures when cultured under these conditions. When cocultured with HBVPs, *ENG*^+^ EA.hy926 ECs recruit fewer mural cells to these structures, compared with Mock cells (Fig. 3a, b). Moreover, the adhesion of HBVPs to a monolayer of *ENG*^+^ or Mock EA.hy926 ECs was analyzed, and a reduced adhesion to ECs that overexpress endoglin was found (Fig. 3a, c).

We also aimed to confirm whether endoglin overexpression decreases vessel maturation in vivo. We found no differences in the expression of *Pdgfrb* in retinas from WT and *ENG*^+^ mice (Supplementary Fig. 4A), suggesting an equivalent number of mural cells in the retinas. To analyze the distribution of these mural cells, colabeling of the retinal endothelium with FITC-isolectin B4 and NG2, which is a pericyte marker, was performed. There was almost complete colocalization of these two markers in WT retinas, but, in *ENG*^+^ retinas, endothelium that was not entirely covered by mural cells and also pericytes that did not appear to be bound to the endothelium can be observed, suggesting impaired mural cell attachment (Fig. 3d, e). Similarly, we analyzed the expression of mural cell markers in plugs of Matrigel^®^, and we observed the same results as in retinas. It seems that there are equal numbers of mural cells in plugs implanted in WT and in *ENG*^+^ mice (Supplementary Fig. 4B). Then, the vessel maturation state by quantifying the immunofluorescence of the endothelial marker Pecam1 and the mural marker NG2 was analyzed. This assay confirmed that vessels from *ENG*^+^ plugs have less mural coverage than vessels from WT plugs (Fig. 3d, f).

**Fig. 4:**
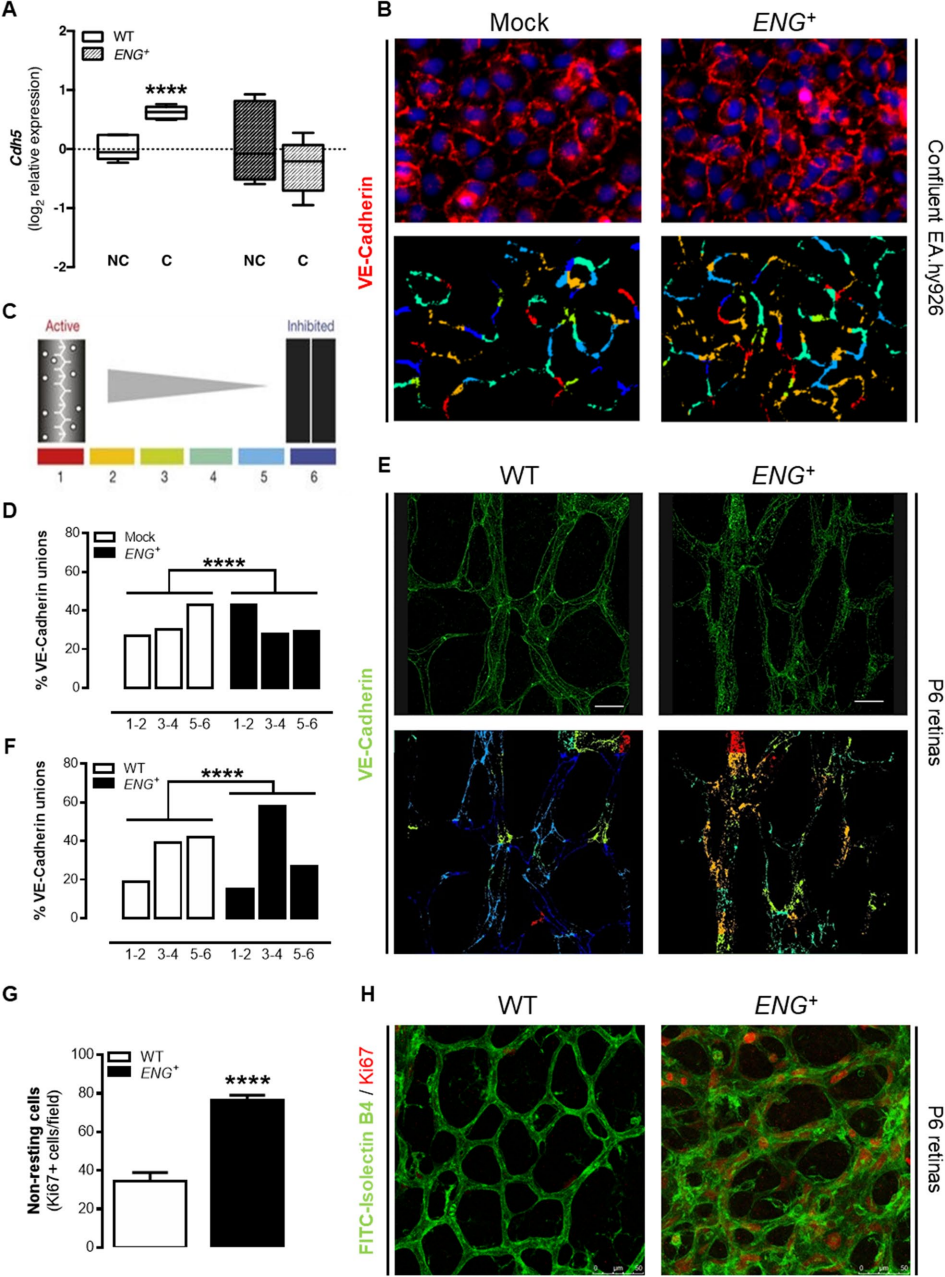
Continuous endoglin overexpression alters endothelium stability in vivo. **a** qPCR analysis of *Cdh5* expression in confluent (C) and nonconfluent (NC) MLECs [*n*(WT) = 6, *n*(*ENG*^+^) = 6; *p* = 0.0237]. **b** Upper panel: VE-cadherin staining in confluent EA.hy926 ECs. Lower panel: pattern of VE-cadherin junctions in confluent EA.hy926 ECs, using the “patch algorithm” in MATLAB™. **c** Schematic illustration of patch classification numbers and colors used to quantify the pattern of VE-cadherin junctions. **d** Quantification of each type of junction in confluent EA.hy926 ECs [*n*(WT) = 10, *n*(*ENG*^+^) = 10; *p* < 0.0001]. **e** Upper panel: VE-cadherin staining in the retinal vasculature of P6 pups. Lower panel: Pattern of VE-cadherin junctions in the retinal vasculature of P6 pups, using the “patch algorithm” in MATLAB™. **f** Quantification of each type of junction in the retinal vasculature of P6 pups [*n*(WT) = 13, *n*(*ENG*^+^) = 12; *p* < 0.0001]. **g** Quantification of non-resting (Ki67^+^) cells in the central area of the retinal vasculature of P6 pups [*n*(WT) = 4, *n*(*ENG*^+^) = 5; *p* < 0.0001]. **h** Ki67 (red) and FITC-lectin (green) staining in the central area of the retinal vasculature of P6 pups

On the other hand, the mural cell coverage of the blood vessels was assessed in the soleus muscle after 14 days of ischemia by immunohistochemistry of α-SMA. At this time point, the vessel coverage is complete in almost all vessels without differences between *ENG*^+^ and WT muscles (Fig. 3g, h). Finally, we analyzed pericyte coverage in retinas from adults and both *ENG*^+^ and WT mice present a complete coverage, without any difference between *ENG*^+^ and WT retinas (Fig. 3i). The absence of an altered phenotype in the adult vasculature is consistent with the normal development of the mice during lifetime.

Thus, in the models in which mural cells recruitment is beginning, this impairment is evident. However, in the models without active angiogenesis vessel coverage seems to be normal. Taking all together, it seems that continuous endoglin overexpression results in a delay of pericyte or smooth muscle cell recruitment.

### Continuous endoglin overexpression prevents VE-cadherin organization and EC resting

We aimed to assess whether altered vascular architecture in mice with constitutive endoglin overexpression is due to an effect on endothelium stability. The expression of VE-cadherin (*Cdh5*) in confluent and nonconfluent MLECs was analyzed. These cells create stable junctions when they are confluent, and *Cdh5* gene expression levels directly affect these junctions [32, 33]. We observed an increase in *Cdh5* expression in confluent WT MLECs compared to the level in nonconfluent cells. This increase was not observed in *ENG*^+^ MLECs (Fig. 4a), suggesting a reduction in endothelium stabilization in vitro. We also analyzed the gene expression levels of *Cdh5* and the angiopoietin receptor Tie2 (*Tek*), whose levels are increased in the quiescent endothelium [34], in retinas and plugs of Matrigel^®^. There was a decrease in the level of *Tek* expression in *ENG*^+^ retinas compared to the level in WT retinas, although no differences in *Cdh5* expression were found (Supplementary Fig. 4C). No differences were also found in the expression of these genes in Matrigel^®^ plugs (Supplementary Fig. 4D).

However, the role of VE-cadherin in endothelium stabilization depends not only on its expression but also on its distribution along the cell membrane. After the first contacts between ECs, a punctuated pattern of VE-cadherin can be observed. These junctions are called discontinuous, irregular or active and characterize the response to stimuli that reduce the integrity of the endothelial barrier. Subsequently, these complexes create the continuous, regular or inactive adhesions that are present in quiescent vessels [35]. For this reason, the VE-cadherin distribution by immunofluorescence in confluent ECs was analyzed. Confluency should inhibit cell proliferation and stimulate VE-cadherin organization in stable junctions. The image quantification using the “patching algorithm” for MATLAB software [36] demonstrated that *ENG*^+^ cells have a greater number of irregular junctions, compared with Mock cells (Fig. 4b–d). Moreover, the VE-cadherin distribution was studied by immunofluorescence in the central area of the retinal vasculature, because under physiological conditions that should be a stabilized area. Similarly, *ENG*^+^ retinas have more irregular junctions than WT retinas (Fig. 4e, f).

Additionally, we studied the lack of stabilization in *ENG*^+^ endothelium measuring the presence of Ki67-positive cells in this central area of the retinal vasculature. As shown in Fig. 2k, endoglin expression in the central area of the retinal vasculature is almost undetectable in WT retinas because of the physiological downregulation of endoglin. However, in *ENG*^+^ retinas, since the promotor for human endoglin is not regulated, the expression of endoglin is equivalent to endoglin expression in the angiogenic front of WT retinas. Thus, *ENG*^+^ mice are a good model to analyze the effect of a lack of a downregulation of endoglin levels during angiogenesis. Immunofluorescence of Ki67 showed that the *ENG*^+^ retinal vascular plexus contains more non-resting ECs (Fig. 4g, h).

Altogether, these results confirm that continuous endoglin overexpression prevents endothelium stabilization and VE-cadherin organization.

### Continuous endoglin overexpression produces leaky vessels that facilitate tumor cell intravasation and metastasis generation

**Fig. 5:**
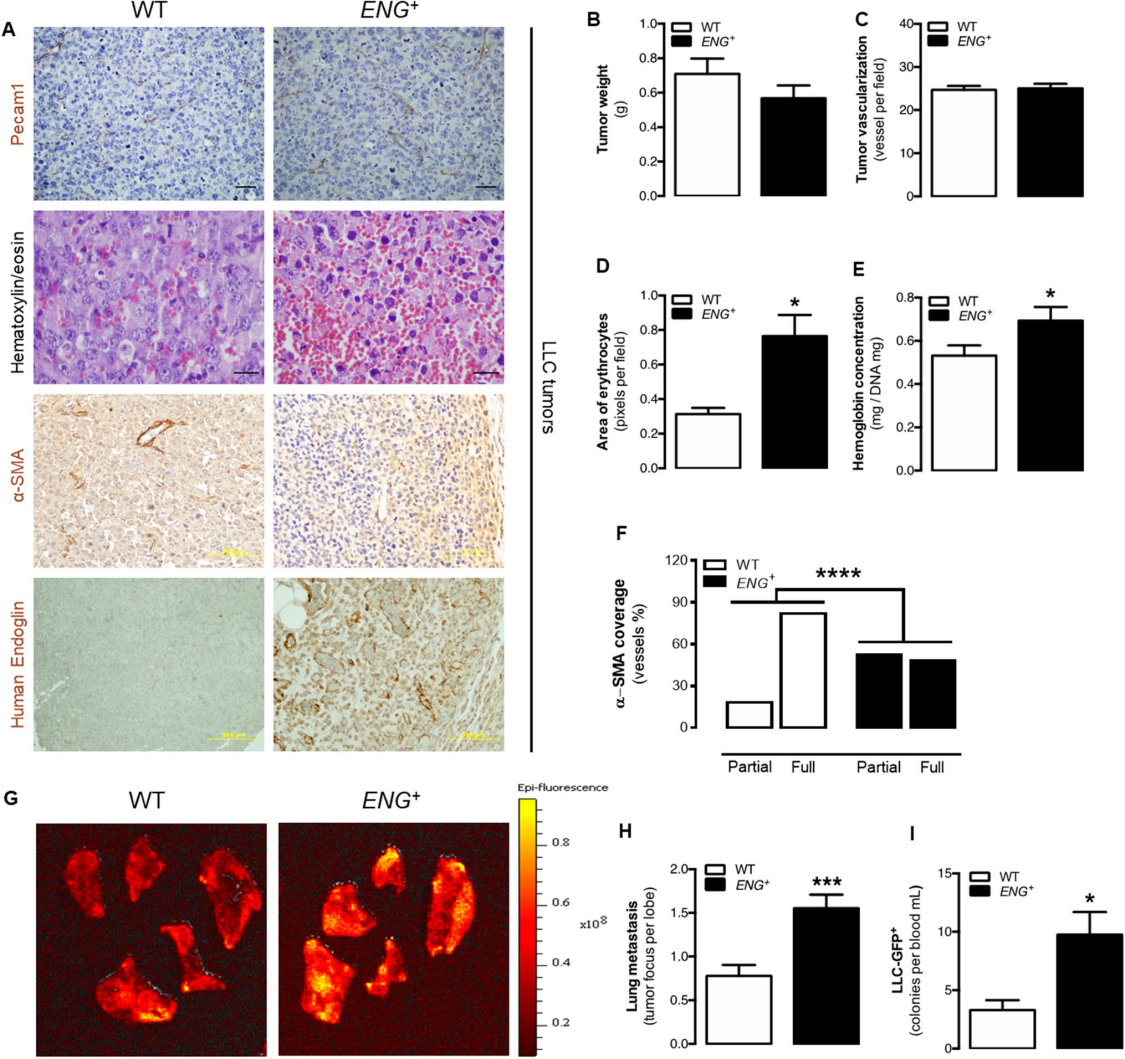
Permanent endoglin overexpression does not increase growth and vascularization in tumors but prevent vessel maturation and facilitates tumor cell metastasis. **a** First panel: Pecam1 immunostaining in the tumor tissue showing tumor vessels. Second panel: Hematoxylin–eosin staining of tumor tissue showing characteristic blood extravasation, blood lakes and edema. Third panel: α-SMA immunostaining in the tumor tissue showing vessel maturation. Fourth panel: human endoglin immunostaining in the tumor tissue. **b** Weight of tumors implanted in mice after 10 days [*n*(WT) = 35, *n*(*ENG*^+^) = 26; *p* = 0.3329]. **c** Quantification of the number of Pecam1-positive vessels in tumor tissue [*n*(WT) = 4, *n*(*ENG*^+^) = 6; *p* = 0.8758]. **d** Quantification of the area filled by erythrocytes in the tumor revealed by hematoxylin–eosin staining [*n*(WT) = 4, *n*(*ENG*^+^) = 6; *p* = 0.0484]. **e** Quantification of hemoglobin concentration in the tumor tissue [*n*(WT) = 30, *n*(*ENG*^+^) = 21; *p* = 0.0427]. **f** Quantification of the ratio of vessels in the tumor tissue that is partially or totally covered by α-SMA immunostained tissue [*n*(WT) = 8, *n*(*ENG*^+^) = 13; *p* < 0.0001]. **g** Epi-fluorescence images of mouse lung lobe metastases of LLC-GFP^+^ tumor cells. **h** Quantification of tumor metastatic foci per mouse lung lobe [*n*(WT) = 8, *n*(*ENG*^+^) = 9; *p* = 0.0004]. **i** Quantification of circulating LLC-GFP^+^ tumor cells in mice [*n*(WT) = 8, *n*(*ENG*^+^) = 9; *p* = 0.0107]

Subcutaneous injection of Lewis lung carcinoma (LLC) cells in *ENG*^+^ and WT mice results in the generation of solid tumors in the flanks of the mice. Our first hypothesis was that endoglin overexpression will result in increased tumor vascularization and growth. However, consistently with the results exposed in this work, the tumors that developed in *ENG*^+^ mice did not grow larger than those in the WT mice as no significant differences were found between the weight of the tumors in the two mouse lines (Fig. 5b). Moreover, immunohistochemistry of Pecam1 did not show differences in the number of vessels generated inside the tumors (Fig. 5a, c). However, hematoxylin/eosin staining showed that the vessels inside the tumors developed in *ENG*^+^ mice were even more impaired and leaky than those that developed in WT mice, resulting in increased blood extravasation, blood islets and tumor edema (Fig. 5a, d). This result was confirmed by measuring the hemoglobin concentration in the tumor tissue, which was higher in the tumors from *ENG*^+^ than in those from WT mice (Fig. 5e). We have above described that continuous endoglin overexpression leads to a delayed endothelium stability and maturation that, in a context of continuous angiogenesis as in tumors, could result in a reduced and impaired vessel maturation. To confirm this hypothesis, immunohistological analysis of mural cells recruitment by α-SMA staining was performed. As tumoral vascularization is a dysregulated process, it is frequent the presence of blood vessels with incomplete or partial coverage with mural cells. This kind of vessels are more frequent in tumors implanted in *ENG*^+^ mice, in which vessels are formed by ECs with endoglin overexpression, than in those from WT mice (Fig. 5a, f).

The lower stability of the endothelium and the impaired vessel maturation can lead to an enhanced permeability of the vessels. Therefore, the evidence gathered so far may explain the increased infiltration of erythrocytes in the stroma of tumors developed in *ENG*^+^ mice (Fig. 5a, d). Interestingly, all the vessel structure alterations observed after tumor angiogenesis could have more serious consequences, such as the intravasation of tumor cells. For this reason, the presence of metastases in WT and *ENG*^+^ mice after tumor induction with LLC cells infected with green fluorescence protein (GFP) was analyzed. Mouse lungs were analyzed to quantify the number of GFP-positive foci as evidence of the presence of tumor cells after metastasis. In agreement with the previous results, compared with WT lungs, *ENG*^+^ lungs had more tumor foci (Fig. 5g, h). Moreover, compared with WT, *ENG*^+^ mice had a higher number of circulating GFP-LLC cells, as evidenced by the fact that blood from these mice generated more GFP^+^ cell colonies in vitro (Fig. 5i). These results suggest that continuous endoglin overexpression promotes tumor cell intravasation and the development of metastases in mice.

## Discussion

In this study, we demonstrate that sustained high levels of endoglin do not improve angiogenesis; instead, constitutive endoglin overexpression produces alterations in the structure of the vasculature. These alterations do not seem to be due to defects during the first phases of angiogenesis but endoglin overexpression alters vessel stabilization and maturation, as shown in vitro and in vivo. In tumors, this instability leads to the extravasation of erythrocytes to the tumor stroma and the intravasation of malignant cells, without affecting the tumor size. All this may explain the mechanism, which has never been demonstrated before, by which high levels of endoglin in different solid tumors are associated with a worse prognosis [19, 20] and the mechanism by which anti-endoglin therapy reduces the generation of metastases [24, 25].

Different authors have proposed that because endoglin deficiency impairs angiogenesis [7, 10, 12], its pathological overexpression may enhance it. However, this hypothesis has never been fully demonstrated. We first analyzed the effect of endoglin overexpression on EC proliferation and migration, two physiological processes directly implicated in sprouting of the new vessel. Our results show that endoglin overexpression seems to potentiate a tip-like phenotype, with increased migration and reduced proliferation, but without affecting the overall sprouting. These results agree with those of Jin et al*.* Although they neither found differences in sprouting, they described that, in a mosaic model of *Eng*-knockout and *ENG*-overexpressing ECs, the loss of function of endoglin resulted in reduced tip cell potential whereas human endoglin overexpressing cells where overrepresented in the tip cell position [8]. Consistently, our analysis of human endoglin expression in the *ENG*^+^ pup retinas shows that all ECs express the transgene, but its expression is higher in tip cells.

On the other hand, our results show that in WT retinas endoglin expression is increased in the angiogenic front but it is downregulated in the central zone, consistent with published data [6–8]. Jin et al. described that endoglin downregulation in tip cells may be necessary to limit sprout elongation and to facilitate anastomosis [8]. Similarly, we demonstrate that endoglin downregulation is required to inactivate stalk EC proliferation and initiate stabilization processes like VE-cadherin organization and mural cell recruitment.

Angiogenesis is a complex process that involves more elements than simply the effect of EC on sprouting. For that reason, we analyze the overall process by different in vivo approximations. Our results, not only do not show a greater or better angiogenesis, as was supposed from works in which endoglin-deficit models are used [7, 10, 12], but also reveal that endoglin overexpression affect the angiogenic process.

Since the first phases of angiogenesis does not seem to be affected, we analyzed the maturation phase. Vessel stabilization and maturation requires ECs to start a transition from an active and proliferative phenotype to a quiescent state. Our results show that continuous endoglin expression impaired this process keeping ECs in an active phenotype that present more active VE-cadherin junctions and less pericyte recruitment. Previously, endoglin deficiency had been related to a lower mural cell recruitment [29, 30], and it has been demonstrated that the endoglin extracellular domain participates in the adhesion of the pericytes to the endothelium [31], so it could be thought that endoglin overexpression increased this process. However, our hypothesis is that endoglin overexpression is affecting to pericyte-EC dialog that would result in less pericyte recruitment. Moreover, one of the limitations of our model is that endoglin overexpression occurs ubiquitously, so the increase of endoglin levels in the mural cells could be affecting this process. However, as we also observed this lower pericyte recruitment and adhesion in in vitro models, in which only ECs overexpress endoglin, we hypothesized that the alterations observed in vivo are due, at least partially, to the effect of endoglin on ECs. In the long term, other mechanisms should be involved since this phenotype is only present in a context of active angiogenesis, given that adult vasculature seems normal.

**Fig. 6:**
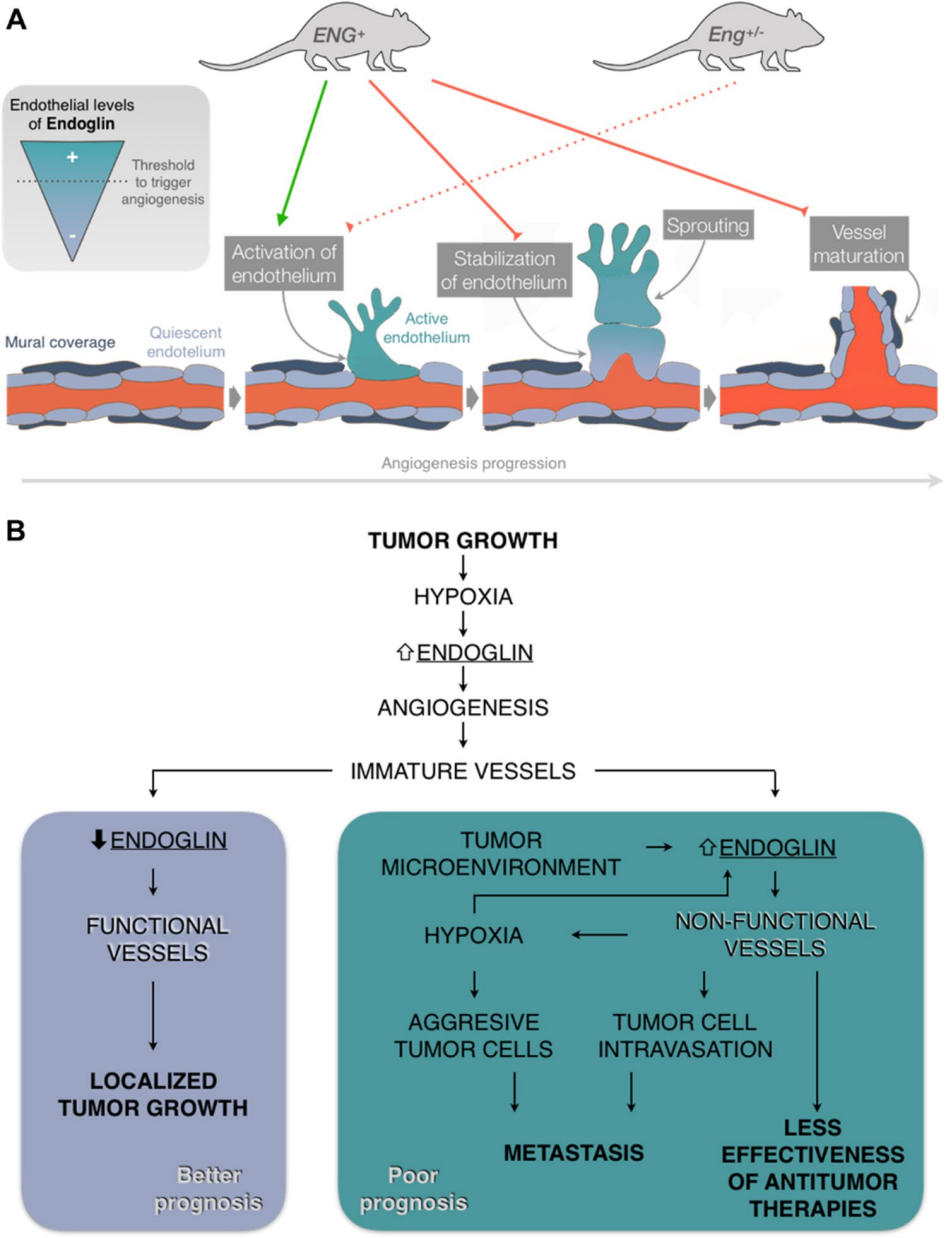
Models of endothelial endoglin regulation during physiological and tumor angiogenesis. **a** Endoglin levels in the endothelium need to be carefully regulated for the correct activation (upregulation) and subsequent stabilization (downregulation to basal levels) of the newly formed vessel. Consistent endoglin overexpression (*ENG*^+^) promotes endothelium activation but impedes vessel stabilization and maturation. A lack of endoglin (*Eng*^+/−^) may prevent proper activation of the endothelium and triggering of angiogenesis. **b** Tumoral continuous endoglin overexpression could difficult vessel normalization which reduces the effectiveness of anti-tumor therapies, enhances the aggressiveness of tumor cells by further increasing hypoxia and promotes the appearance of metastases. On the contrary, downregulation of endoglin after tumor angiogenesis promotes vessel stabilization and thus tumor growth control, which could explain the positive effect of anti-endoglin therapies in cancer control

Thus, considering the results of previous studies and our results, we propose a model that could explain the consequences of endoglin levels being above or below physiological levels (Fig. 6a) and the importance of fine-tuned endoglin regulation during angiogenesis. According to this model, it is necessary for endoglin expression to increase during sprouting [6–8] up to a threshold level, which may not be reached in *Eng*^−/−^ and *Eng*^+/−^ mice, in which the initial phases of angiogenesis do not occur correctly. This is supported by the results of numerous studies that have demonstrated that endoglin expression is increased in the angiogenic edge [6, 7] and that its deficiency can alter the proliferation of ECs [7, 37, 38], migration of ECs [7, 10, 39] and three-dimensional organization of ECs in Matrigel^®^ [7, 10]. These alterations may lead to impaired sprouting from *Eng*^+/−^ aortic rings [7] and finally to defects in vessel formation in *Eng*^+/−^ animals [7, 10, 12]. On the other hand, our results suggest that physiological endoglin expression is enough to develop the initial phases of angiogenesis, as endoglin overexpression does not enhance these phases. In contrast, constitutive endoglin overexpression alters endothelium stabilization and mural cell recruitment, as demonstrated in this study, both in vitro and in vivo. Thus, during the final phases, it seems necessary for endoglin levels to decrease to basal levels to allow the maturation of the vessels, as sustained endoglin overexpression maintains the activation of ECs. In fact, Jin et al*.* draw a conclusion in the same line as this model. These authors, like us, observe that the expression of endoglin is high in the vascular front of the retinas of WT pups, but not in the tip cells, and conclude that the expression of endoglin in these cells is downregulated to allow begin the anastomosis and the resolution of angiogenesis [8]. Some authors have shown that endoglin-deficient vessels also present altered stabilization and maturation [31, 40], giving rise to more permeable vessels [31, 41, 42]. However, this phenotype seems to be explained by the lack of direct interaction between endothelial endoglin and the integrins of pericytes [31].

Regarding cancer development, other authors have shown that endoglin deficiency or different anti-endoglin treatments reduce the vascularity of tumors and, therefore, decrease tumor growth [23, 43–46]. However, according to our model and our results, the poor prognosis associated with high levels of endoglin is not related to a greater number of vessels or a larger tumor size. Instead, we propose a second model (Fig. 6b) in which the lack of downregulation of endoglin levels in the tumor endothelium after angiogenesis leads to the lack of stabilization and increased impairment of the function of tumor vessels. This could result in an inadequate nourishment of the tumor, limiting its growth but also increasing hypoxia, which causes tumor cells to acquire a more aggressive phenotype [47, 48]. In addition, the intravasation of malignant cells could be facilitated by increased vessel instability and impaired mural coverage [49]. Accordingly, our results show increased tumor cell intravasation in *ENG*^+^ mice compared with WT mice. Considering that metastases are responsible for 90% of cancer deaths, we propose the following new hypothesis: the worse prognosis of patients with tumors with high levels of endoglin is not due to increased tumor size; instead, the overexpression of endoglin maintains the activation of the endothelium and increases the risk of metastases. This hypothesis is in accordance with previous observations of increased metastases in tumors with high levels of endoglin staining [50].

As stated previously, some studies have shown that the administration of anti-endoglin antibodies, such as TRC105, decreases the number of metastases [24, 25], although an explanation of the mechanisms by which endoglin contributes to metastasis generation has been lacking. Considering our model (Fig. 6b), we hypothesize that anti-endoglin therapies are not only antiangiogenic, as described above [23, 51], but also reduce the generation of metastases by promoting the stabilization of the endothelium and the normalization of vessels. One limitation of our study, as we have mentioned previously, is that endoglin overexpression also occurs in all cell types other than ECs, including those in the tumor microenvironment, which may also play an important role in the malignancy of tumors. For example, beyond its antiangiogenic effect on the endothelium, anti-endoglin treatment inhibits cancer-associated fibroblast (CAF) invasion and metastasis [52]. However, again we think that our in vitro results about permanent activation of EC and pericyte recruitment can explain, at least partially, what we observed in vivo. Therefore, although it may not be the only cell type involved, we can conclude that the persistence of active endothelium is one of the main causes of the intravasation of tumor cells and the generation of metastases.

Moreover, several studies have shown that the low functionality of tumor vessels prevents conventional anti-tumor therapies from reaching the entire tumor, reducing their effectiveness [47]. This is also likely to occur in tumors with high levels of endoglin and may also be related to a worse prognosis (Fig. 6b).

In summary, we demonstrate that, although an increase in endoglin expression is necessary during the first phases of angiogenesis, an alteration in endoglin downregulation to basal levels may alter vascular stabilization and mural cell recruitment. This is a key event in tumors, in which the proangiogenic stimuli are very potent and vessel instability facilitates the intravasation of malignant cells and hampers anti-tumor therapy efficacy. Therefore, the normalization of the vasculature resulting from the administration of anti-endoglin therapies may reduce tumor cell intravasation and increase the effectiveness of chemotherapy, as has already been observed with anti-VEGF and ALK1-Fc treatments [47, 53, 54].

## Electronic supplementary material

Below is the link to the electronic supplementary material.

Supplementary file1 (PDF 883 kb)
