# Supplementary material for: Continuous endoglin (CD105) overexpression disrupts angiogenesis and facilitates tumor cell metastasis
Source: Angiogenesis. 2020 Jan 3;23(2):231–47. doi: 10.1007/s10456-019-09703-y (PMC7160077; doi:10.1007/s10456-019-09703-y)
Supplement: Supplementary file 1 — Supplementary file1 (PDF 883 kb) [file 10456_2019_9703_MOESM1_ESM.pdf]

SUPPLEMENTARY FIGURES

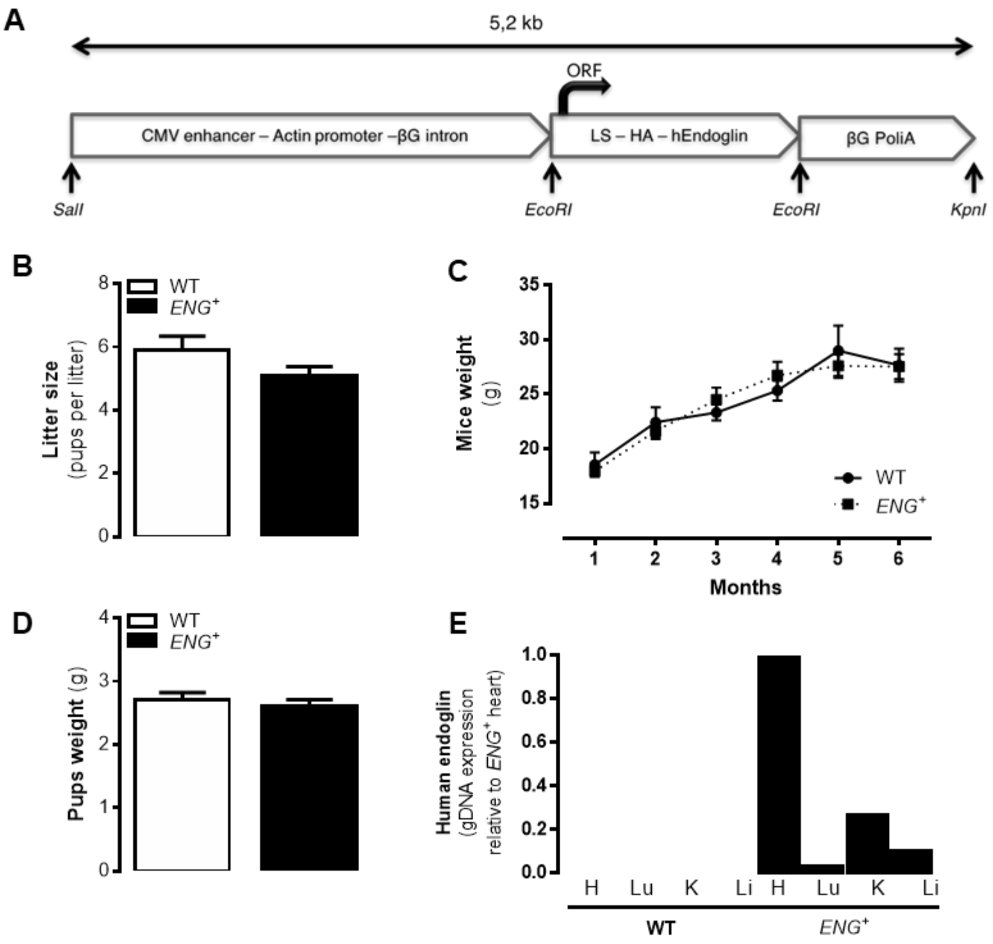

Supplementary Fig. 1. Description of *ENG⁺* mice. (A) Structure of the vector containing the human endoglin gene used for the stable infection of EA.hy926 cells to generate endoglin overexpression (*ENG⁺*) and *ENG⁺* mice (MLEC source). The same vector without the endoglin gene was used to generate *Mock* EA.hy926 cells. (B) Litter size obtained at the crosses of WT and *ENG⁺* mice [n(WT)=24, n(*ENG⁺*)=62; p=0.1234] (C) Mice weight of the WT and *ENG⁺* lines during its growth [n(WT)=9, n(*ENG⁺*)=9; p=0.9357]. (D) Weight of WT and *ENG⁺* 6 days-old pups [n(WT)=16, n(*ENG⁺*)=13; p=0.2983]. (E) qPCR detection of human endoglin expression in WT and *ENG⁺* hearts (H), lungs (Lu), kidneys (K) and Liver (L).

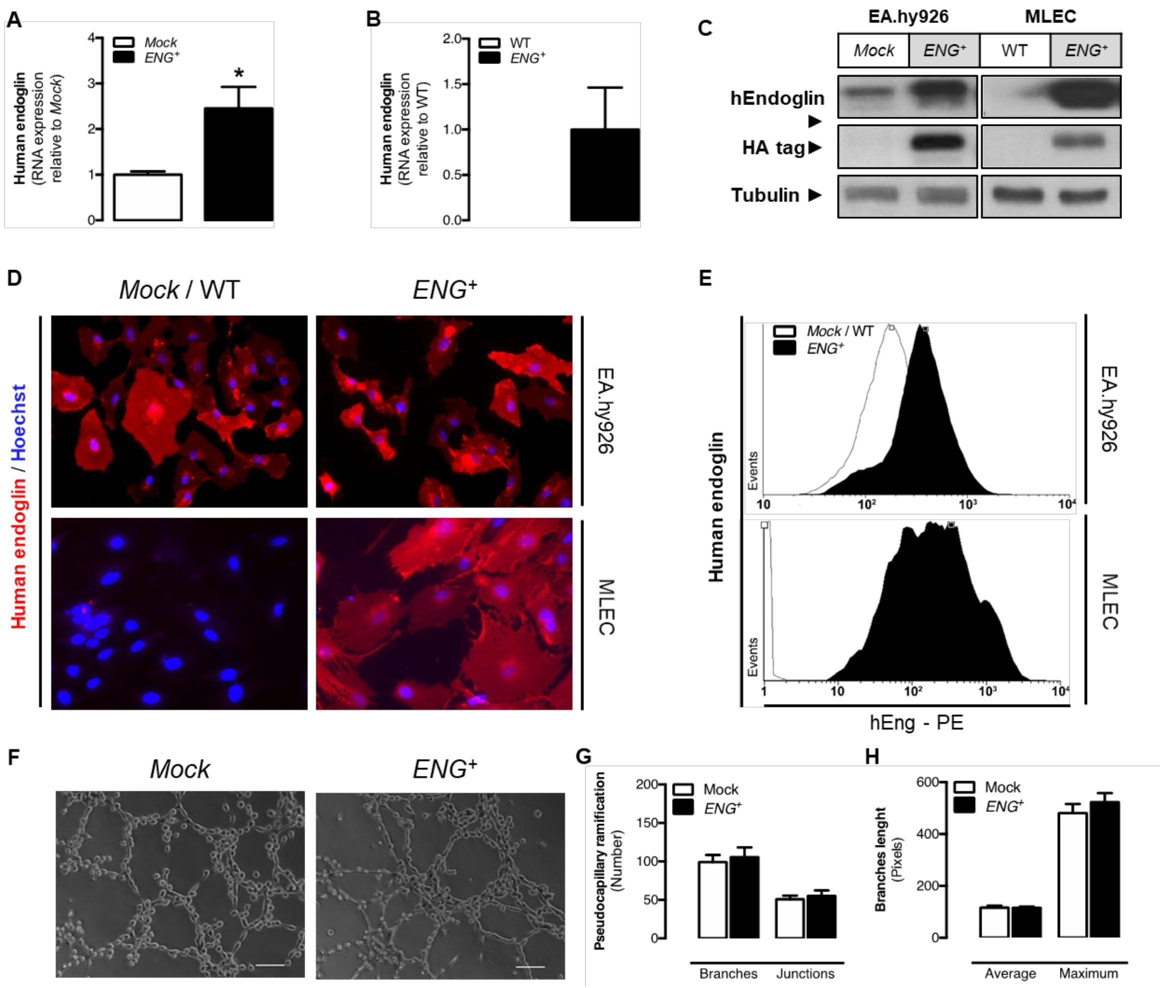

Supplementary Fig. 2. Characterization of transgenic ECs overexpressing endoglin. (A) qPCR detection of human endoglin expression in *Mock* and *ENG*$^+$ EA.hy926 cells. (B) qPCR detection of human endoglin expression in WT and *ENG*$^+$ MLEC cells. (C) Western blot assessment of human endoglin in the lysates of EA.hy926 and MLEC cells. (D) Immunofluorescence of human endoglin in EA.hy926 and MLEC cells. (E) FACS measurement of surface human endoglin in EA.hy926 and MLEC cells. (F) Pseudocapillary-like structures formed by *Mock* and *ENG*$^+$ EA.hy926 cells in Matrigel®. (G) Quantification of the number of branches and junctions of the EA.hy926 pseudocapillary-like structures [n(Mock)=3, n(*ENG*$^+$)=3; p(branches)=0.6819, p(junctions)=0.6164]. (H) Quantification of the average and maximum lengths of the branches of the EA.hy926 pseudocapillary-like structures [n(Mock)=3, n(*ENG*$^+$)=3; p(average)=0.9536, p(maximum)=0.4029].

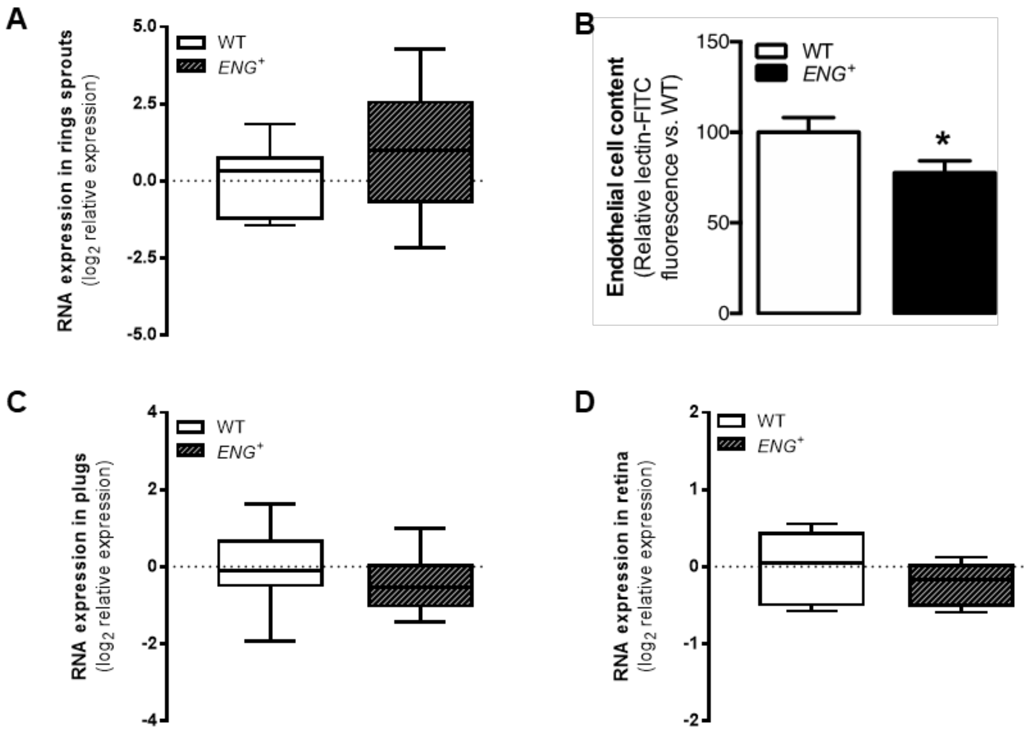

Supplementary Fig. 3. The effect of permanent endoglin overexpression on *ex vivo* sprouting and *in vivo* angiogenesis. (A) qPCR analysis of *Pecam1* expression in sprouts from aortic rings [n(WT)=10, n(ENG^+)=10; p=0.1960]. (B) Quantification of DIVAA EC content, measured by the FITC-lectin signal 9 days after implantation [n(WT)=12, n(ENG^+)=11; p=0.0479]. (C) qPCR analysis of *Pecam1* expression in plugs of Matrigel® [n(WT)=11, n(ENG^+)=9; p=0.2980]. (D) qPCR analysis of *Pecam1* expression in the retinas of P6 mouse pups [n(WT)=6, n(ENG^+)=6; p=0.3785].

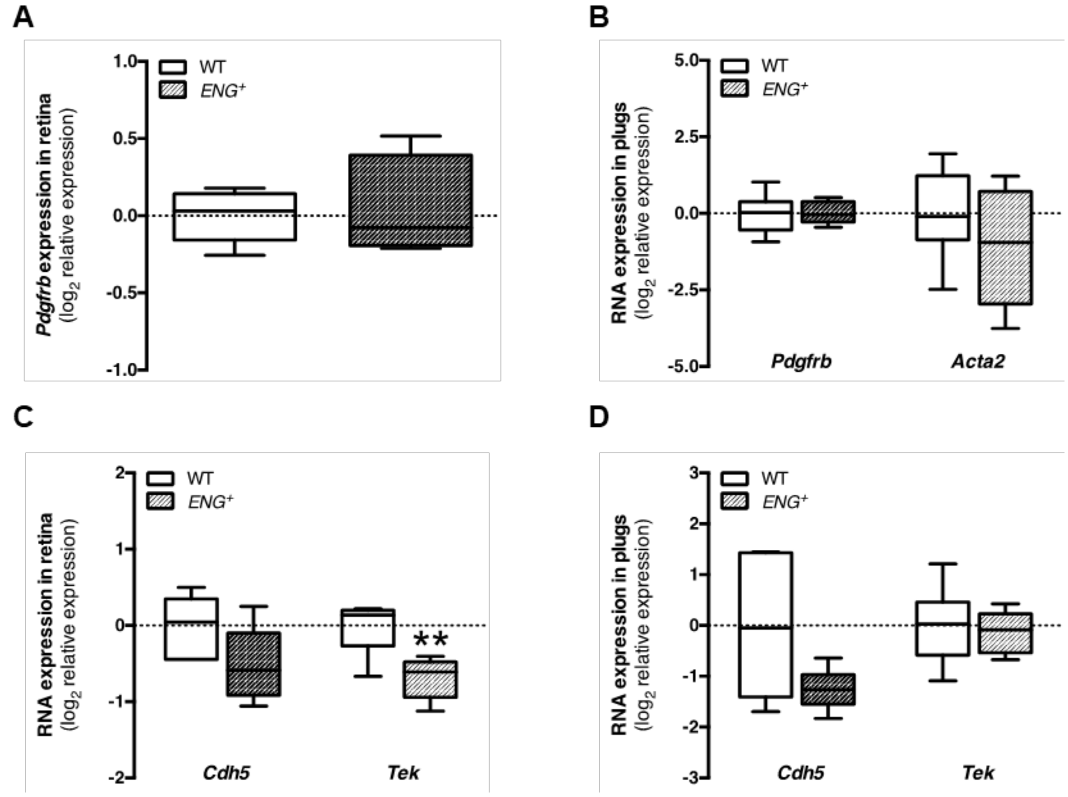

Supplementary Fig. 4. The effect of permanent endoglin overexpression on endothelium stabilization and mural cell coverage. (A) qPCR analysis of *Pdgfrb* expression in the retinas of p6 mouse pups [n(WT)=6, n(*ENG*$^{+}$)=6; p=0.6977]. (B) qPCR analysis of *Pdgfrb* and *Acta2* expression in plugs of Matrigel® [n(WT)=9, n(*ENG*$^{+}$)=8; p(*Pdgfrb*)=0.9065, p(*Acta2*)=0.2327]. (C) qPCR analysis of *Cdh5* and *Tek* expression in the retinas of p6 mouse pups [n(WT)=6, n(*ENG*$^{+}$)=6; p(*Cdh5*)=0.0733, p(*Tek*)=0.0061]. (D) qPCR analysis of *Cdh5* and *Tek* expression in plugs of Matrigel® [n(WT)=9, n(*ENG*$^{+}$)=8; p(*Cdh5*)=0.0684, p(*Tek*)=0.6843].

SUPPLEMENTARY METHODS

Mouse Lung Endothelial Cell (MLEC) culture (dx.doi.org/10.17504/protocols.io.28ighue): Lungs from three mice (3-months old or younger) were removed, washed and mechanically disaggregated. The homogenate was incubated in 500 µg/mL collagenase (Sigma-Aldrich) and 60 µg/mL DNase (Sigma-Aldrich) solution at 37 °C for 1 hour. The solution was filtered through a 70-micron strainer. After 48 hours in culture, the macrophages and monocytes were removed using a specific antibody against CD16/CD32 and magnetically labeled secondary antibodies (BD Biosciences). Forty-eight hours later, the ECs were selected using specific antibodies against CD102 and magnetically labeled secondary antibodies (BD Biosciences). The labeled cells were collected and cultured.

Cellular infection (dx.doi.org/10.17504/protocols.io.23mggk6): a 293T virus packaging cell line was transfected with a vector containing the human endoglin cDNA sequence or GFP sequence, resulting in the packaging and production of viruses carrying the sequences. The viral supernatant was collected and used to culture EA.hy926 EC and LLC cells, respectively. Cells positively infected were selected by puromycin resistance.

Direct cell proliferation (dx.doi.org/10.17504/protocols.io.28xghxn): Two 60 mm-culture dishes were with $7x10^4$ cells. After 8 hours of culture, one dish was trypsinized and cells were counted (control count) using a Countess® Automated Cell Counter (Thermo Fisher Scientific). The second dish was cultured for 72 hours, and then trypsinized and cells were counted. Cell count was expressed in relation to control count.

Wound healing migration assay (Scratch assay) (dx.doi.org/10.17504/protocols.io.28yghxw) $3x10^5$ EA.hy926 ECs were plated in 6-well plates and grown to 100% confluence. Then, scratches were generated with a 2-µL pipette tip, and fresh culture medium was added. Microscopy photographs were taken every 30 minutes for 20 hours. The timepoint before wound closure was selected (14.5 hours), and the distance separating cell flanks was measured and subtracted from the initial distance at 10 different points per image.

Transwell assay (dx.doi.org/10.17504/protocols.io.28zghx6): MLECs and EA.hy926 cell migration towards and invasion through Matrigel® towards VEGF was studied with

porous transwells with Fluoroblock® (Corning). First, 6x10^4 cells were labeled with calcein-AM prior to culture in the transwell, which was either uncoated or Matrigel®-coated. Gradients of FBS and VEGF were created between the upper and lower chambers to stimulate cells to migrate towards the lower chamber. The increase in fluorescence was measured in the lower chamber using a FluoroSkan Ascent System.

Capillary-like structures in Matrigel® (dx.doi.org/10.17504/protocols.io.28ughww): Twenty-four-well plates were coated with Matrigel® and allowed to polymerize in the incubator at 37 °C for 30 minutes. Then, 1.5x10^5 EA.hy926 ECs were resuspended in DMEM with 0% FBS and plated on the Matrigel®. After 3 hours at 37 °C, tube formation was visualized using an Axiovert 200M fluorescence microscope. Photographs were taken of three different fields from each well. The number and length of branches were analyzed with Fiji software.

Co-culture of ECs and pericytes in Matrigel® (dx.doi.org/10.17504/protocols.io.28vghw6): 1.5x105 EA.hy926 were stained with CellTrackerTM Orange CMRA Dye (Thermo Fisher Scientific), and 7.5x104 HBVPs were stained with calcein-AM, following the manufacturer's indications. Then, EA.hy926 and HBVPs were mixed and plated on Matrigel®. After 3 hours at 37 °C, tube formation was visualized using an Axiovert 200M fluorescence microscope. Photographs were taken of three different fields from each well. The number and length of branches and the areas of the ECs and pericytes were analyzed with Fiji software.

Pericyte adhesion (dx.doi.org/10.17504/protocols.io.28wghxe): 6x104 EA.hy926 ECs were plated on 24-well plates and grown to confluence. Then, 8x104 HBVPs were stained with calcein-AM and seeded on the EC monolayer previously stained with CellTrackerTM Orange CMRA Dye. Adhesion was allowed to progress for 2 hours. Non-adherent pericytes were removed through two washes with PBS. HBVP fluorescence was measured at the baseline and after the washes with a FluoroSkan Ascent System.

Aortic ring assay (dx.doi.org/10.17504/protocols.io.28sghwe): Mice were humanely euthanized, and the thoracic cavity was exposed. The thoracic aorta was dissected, isolated and collected in Ham's F-12 cold washing medium (Thermo Fisher Scientific). Then, 1-mm thick aortic rings were cut and incubated in harvesting DMEM at 37 °C and

5% CO_2 for 12 to 16 hours. Following incubation, each aortic ring was cultured in 30 µL of Matrigel® in MLEC medium (described in the section 'Cells and cell culture'). The aortic rings were incubated at 37 °C and 5% CO_2 for 5 days, during which time the sprouting from the ring endothelium takes places, prior to confocal image acquisition or RNA isolation. For confocal microscopy, the sprouts were stained with calcein-AM (Sigma-Aldrich) and examined (Leica SP5). Sprout volume was quantified using Fiji software. For the gene expression assays, RNA was pooled from 4-6 rings from the same mouse.

Mouse hindlimb ischemia (dx.doi.org/10.17504/protocols.io.28nghve): The basal perfusion levels of previously shaved lower limbs were measured with a Doppler Laser Moor LDLS (Moor Instruments). Under anesthesia, the left lower hindlimb was operated on, and the femoral artery was ligated with 5.0 non-absorbent silk. The skin was closed with interrupted sutures. Perfusion was evaluated by laser Doppler flowmetry on days 1, 3, 5, 7, 14, 21 and 28 after ischemia by comparison with the contralateral nonligated limb.

Direct *In Vivo* Angiogenesis Assay (DIVAA™) (dx.doi.org/10.17504/protocols.io.28pghvn): Silicone cylinders closed at one end were filled with 25 µL of Matrigel® premixed with the following angiogenesis modulating factors: 3 ng/µL VEGF (R&D Systems), 9 ng/µL bFGF (R&D Systems) and 2 ng/µL bovine heparin (Sigma-Aldrich). These angioreactors were then implanted subcutaneously in the flanks of mice. Nine days postimplantation, the tubes were removed from the anesthetized animals, and the length of the reddish-colored zone was measured. Angioreactor content was then collected and disaggregated in dispase (Corning). ECs within the tube were labeled using FITC-lectin. Fluorescence was measured using a FluoroSkan Ascent System (Thermo Fisher Scientific).

Plugs of Matrigel® (dx.doi.org/10.17504/protocols.io.28qghvw): A total of 250 µL of Matrigel® supplemented with 1 ng/µL VEGF, 1 ng/µL bFGF and 1 ng/µL bovine heparin was directly injected into the flank of each mouse. After 7 days, the mice were humanely euthanized, and the plug was isolated and stored at -80 °C for RNA extraction or in Somogyi solution (1X PBS, 4% PFA and 0.25% picric acid) for 24 hours at 4 °C for histological analysis.

Retina dissection and immunostaining (dx.doi.org/10.17504/protocols.io.28rghv6): P6 and P17 pups were humanely euthanized, and their eyes were enucleated and either used for RNA isolation or fixed in 4% PFA in 2X PBS for 10 minutes. Fixed eyes were transferred into cold 2X PBS until dissection. For retina isolation, the cornea, iris, vitreous humor and hyaloid vessels were removed and discarded, and the retina was dissected from the sclera. Retinas were washed in PBS, and 4 radial incisions were made. Retinas were slowly pipetted into cold methanol at least 20 minutes before staining. Retinas were blocked in blocking solution (0,3% Triton X-100, 0,5% Tween-20, 0,2% BSA and 5% donkey or goat serum in PBS 2X) for 1 h at room temperature. Then, primary antibody was incubated overnight at 4°C and secondary antibody for 4 h at room temperature. FITC-Lectin was incubated at the same time as the secondary antibody. Retinas were mount on a slide using a drop of ProLong^{TM} Gold Antifade Mountant as mounting medium.

LLC cell tumor xenograft model (dx.doi.org/10.17504/protocols.io.ykkfuuw): LLC cells or GFP-LLC cells (10^6) were resuspended in 50 µL of Matrigel® (Corning) and subcutaneously inoculated to generate each tumor. Two different tumors were induced in the dorsum of each mouse. Ten days later, the mice were humanely euthanized, and the tumors were carefully removed. In the case of the metastasis assay, the tumors were allowed to grow for 14 days. All tumors were weighed and frozen in liquid nitrogen or fixed in 10% paraformaldehyde (PFA) depending on the subsequent analysis.

Tumor hemoglobin and DNA measurement (dx.doi.org/10.17504/protocols.io.28jghun): 100 mg of frozen tumor tissue was homogenized in 1 mL of Drabkin's solution, which was composed of Drabkin's reagent (Sigma-Aldrich) and Brij L23® solution (Sigma-Aldrich). The homogenate was centrifuged at 14,600 *g* for 15 minutes, and the absorbance of the supernatant was measured at 540 nm using an Epoch Microplate Spectrophotometer (BioTek). The hemoglobin concentration was extrapolated from the hemoglobin standard curve (Sigma-Aldrich). For the measurement of the DNA, 50 mg of each tumor was disaggregated by incubation at 56 °C for 4.5 hours in lysis buffer (5 mM Tris (Merck), 5 mM EDTA (Sigma-Aldrich), 100 mM NaCl (Sigma-Aldrich), 1 mM DTT (Sigma-Aldrich), 5 mM spermidine (Sigma-Aldrich) and 250 ng/mL proteinase K (Roche)), with frequent shaking. Proteinase K was inactivated by boiling the samples

for 2 minutes. Then, 1 µg/mL RNase (Roche) was added and incubated at 37 °C for 30 minutes. The DNA concentration was determined in the supernatant using a NanoDrop® ND-1000 spectrophotometer (Thermo Fisher Scientific).

Lung metastasis assay (dx.doi.org/10.17504/protocols.io.28kghuw): Fourteen days after the implantation of GFP-LLC cells, the mice were humanely euthanized, and their lungs were extracted. The lobes were separated and placed in petri dishes. Metastases were visualized using the GFP filter of the bioluminescence equipment *Xeno IVIS® 50* (Caliper Life Sciences) and *Living Image® 3.2* software (Caliper Life Sciences). The number of tumor foci was determined by two different researchers who were blinded to the genotype of each animal. Due to the autofluorescence of the lungs, in each experiment, the lungs of a healthy WT mouse were used as a control for possible false positive foci.

Circulating GFP-LLC assay (dx.doi.org/10.17504/protocols.io.28kghuw)**:** Fourteen days after GFP-LLC implantation, as much blood as possible was extracted from the mice by cardiac puncture. The blood was incubated in erythrocyte lysis buffer (155 mM NH_4Cl, 10 mM $KHCO_3$, 10 mM EDTA) for 10 minutes at 4 °C. Then, it was centrifuged for 3 minutes at 600 g and 4 °C. The resulting pellet was resuspended in DMEM supplemented with 10% FBS and 50 U/mL penicillin-streptomycin and then seeded in a 6-well plate, distributing the volume among all wells. Between 7 and 10 days later, colonies formed by LLC-GFP cells were counted using an *Axiovert 200M* microscope. The number of colonies was corrected for the blood volume extracted.

Western blot (dx.doi.org/10.17504/protocols.io.282ghye): Protein were extracted using lysis buffer (20 mM Tris-HCl, 140 mM NaCl, 10 mM EDTA (pH 8), 1% Igepal CA-630, 10% Glycerol). Total protein lysates (40 µg) were fractioned by SDS-PAGE, transferred onto PDVF membranes (Millipore), blocked in 3% BSA in TTBS and incubated overnight at 4 °C with primary antibodies. Secondary antibodies were incubated for 30-45 minutes at room temperature. Development was performed with a chemiluminescent reagent (0.1 M Tris, pH 9.35 (Merck), 2.2 mM luminol (Sigma-Aldrich) and 2.2 mM p-iodophenol (Sigma-Aldrich), and signals were recorded on X-ray films (Fujifilm).

Flow cytometry (dx.doi.org/10.17504/protocols.io.283ghyn). Cultured cells were harvested and labeled for 15 minutes at room temperature with the monoclonal antibody PE mouse anti-human CD105 (Endoglin) (Ref. 560839, BD Biosciences). After incubation, the samples were washed with PBS and resuspended in 100 µl of PBS Na/K prior to acquisition. In total, 10^4 cells were acquired with the FACSCalibur™ system (BD Biosciences), and the results were analyzed using Infinicyt™ software (Cytognos).

Cellular immunofluorescence (dx.doi.org/10.17504/protocols.io.284ghyw). Cells were plated on sterile glass coverslips and cultured until confluence. The cells were fixed in 4% PFA for 15 minutes and then washed with PBS; PFA was quenched with 4 mM NH_4Cl (Panreac). The coverslips were blocked with 2% BSA (Sigma-Aldrich) and washed with PBST (PBS, 0.05% Tween-20 (Sigma-Aldrich)). The cells were incubated in primary antibody (hybridoma TEA1/58.1 provided by Dr. Sánchez-Madrid, CNIC, Spain) for 3 hours at room temperature. Then, they were incubated with the secondary antibody for 45 minutes. The nuclei were stained using 2 µM Hoechst 33258 (Thermo Fisher Scientific) for a few seconds. The coverslips were mounted on a slide with Prolong® Gold Antifade. The preparations were analyzed using an Axiovert 200M fluorescence microscope.

Table 1. Primers used for cDNA preamplification and qPCR in aortic rings and plugs.

Gene	Preamplication primers (Bio-Rad)	qPCR primers (Bio-Rad)
Pecam1	qMmuCID0005317	qMmuCID0005317
Tek	qMmuCID0015486	qMmuCID0015486
Cdh5	qMmuCID0005343	qMmuCID0005343
Pdgfrb	qMmuCED0045914	qMmuCED0045914
Acta2	qMmuCID0006375	qMmuCID0006375

Table 2. Primer sequences.

Gene	qPCR primers
ENG	AGGTGCTTCTGGTCCTCAGT<br>CCACTCAAGGATCTGGGTCT
Pecam1	AGGACAACCGTACCTTGGGTGACT<br>CAGTTCTGACACGTACCGGGTCTC
Tek	AACAAGAGCGAGTGGACCAT<br>TCCATGGCGCCTTCTACTAC
Cdh5	ATTGGCCTGTGTTTTCGCAC<br>CACAGTGGGGTCATCTGCAT
Pdgfrb	AGGACAACCGTACCTTGGGTGACT<br>CAGTTCTGACACGTACCGGGTCTC
Acta2	AGCCATCTTTCATTGGGATGG<br>CCCCTGACAGGACGTTGTTA
